# Comparison of Different Features and Classifiers for Driver Fatigue Detection Based on a Single EEG Channel

**DOI:** 10.1155/2017/5109530

**Published:** 2017-01-31

**Authors:** Jianfeng Hu

**Affiliations:** Jiangxi University of Technology, Nanchang 330098, China

## Abstract

Driver fatigue has become an important factor to traffic accidents worldwide, and effective detection of driver fatigue has major significance for public health. The purpose method employs entropy measures for feature extraction from a single electroencephalogram (EEG) channel. Four types of entropies measures, sample entropy (SE), fuzzy entropy (FE), approximate entropy (AE), and spectral entropy (PE), were deployed for the analysis of original EEG signal and compared by ten state-of-the-art classifiers. Results indicate that optimal performance of single channel is achieved using a combination of channel CP4, feature FE, and classifier Random Forest (RF). The highest accuracy can be up to 96.6%, which has been able to meet the needs of real applications. The best combination of channel + features + classifier is subject-specific. In this work, the accuracy of FE as the feature is far greater than the Acc of other features. The accuracy using classifier RF is the best, while that of classifier SVM with linear kernel is the worst. The impact of channel selection on the Acc is larger. The performance of various channels is very different.

## 1. Introduction

Traffic accidents are more and more increasing, resulting in a very large number of casualties. Safety driving is fundamental to public health, and fatigue driving can be life threatening. It is crucial and necessary to develop some technologies for detecting driver fatigue [1–3]. There are many methods that have been proposed in the past few years, such as vehicle driving parameters by using various sensors [4], driver behavior characteristics by using video imaging techniques [5, 6], driver physiological parameters by using acquisition and analysis of electrocardiogram (ECG) [7], electrooculogram (EOG) [8], electromyogram (EMG) [9], and EEG [10–12]. As a kind of direct indicator of the brain status, EEG is considered as the “gold” method to identify driver fatigue.

EEG is an objective method for the evaluation of brain state and function, which is often used in auxiliary diagnosis of illness such as epilepsy and seizure. The advantages of EEG are sensitivity for analysis and being relatively cheap for acquisition. Various computational approaches based on EEG signals have been developed for analyzing and detecting driver fatigue.

Fu et al. [13] proposed a fatigue detection model based on Hidden Markov Model and fused physiological and contextual knowledge to assess probabilities of fatigue. They achieved highest accuracy of 92.5% based on EEG signals from two channels (O1 and O2) and other physiological signals. Li et al. [14] collected 16 channels of EEG data and computed 12 types of energy parameters. The number of significant electrodes is reduced using Kernel Principle Component Analysis (KPCA). The experimental results from two channels (FP1 and O1) achieved the highest accuracy of 91.5%. Wali et al. [15] used Discrete Wavelet Transforms to process the EEG signal for fatigue detection and yielded the highest accuracy of 85%. Using Fast Fourier Transform, Simon et al. [16] proposed EEG alpha spindle measures for assessing driver fatigue. Charbonnier et al. [17] made use of the Frobenius distance between the EEG spatial covariance matrices of 6 brain regions, and experimental results had shown that the index based on the alpha band can accurately assess fatigue. Apker et al. [18] predicted driver performance using power spectral density and the linear regression, providing a confidence estimate for the stable driving model. Hajinoroozi et al.'s experimental results showed that channel-wise convolutional neural network achieved robust and improved performance for detection of driver fatigue [19]. Zhao et al. [20] studied an automatic measurement of driving mental fatigue, using a KPCA-SVM classifier and their accuracy was quite high, up to 98.7%. Kong et al. [21] analyzed EEG signals by using Granger-Causality-based brain effective networks and found a significant difference in terms of strength of Granger-Causality in the frequency domain and some changes were more significant over the frontal brain. Zhao et al. [22] observed that coherence was significantly increased in the frontal, central, and temporal brain regions, as well as significant increases in the clustering coefficient and the character path length.

Recently, entropy has been broadly applied in the analysis of EEG signals, considering the fact that it is a complex, unstable, and nonlinear signal [23–28]. Xiong et al. combined features of AE and SE with Support Vector Machine (SVM) classifier to detect driver fatigue, achieving highest accuracy of 91.3% at channel P3 [25]. Chai et al. present independent component by entropy rate bound minimization analysis for the source separate, autoregressive (AR) modeling for the features extraction and Bayesian neural network for the classification algorithm. They achieved an accuracy of 88.2% and the highest value of area under the receiver operating curve (AUC) is 0.93 [26]. Zhang et al. extracted wavelet entropy and SE of EEG and wavelet entropy of EOG and AE of EMG to estimate the driving fatigue stages, and their accuracy was quite high, which is about 96.5%–99.5% using artificial neural network [27]. Kar et al. used five types of entropies, that is, Shannon's entropy, Rényi entropy of order 2, Rényi entropy of order 3, Tsallis wavelet entropy, and Generalized Escort-Tsallis entropy, along with alpha band relative energy for estimation of fatigue level [28]. However, few studies have been conducted for using optimal combination of entropy methods and classifiers based on EEG to study driver fatigue detection.

Multichannels EEG acquisition system, such as the 32-channel EEG system used in my experiment, is relatively complex equipment, which can only be available in laboratories or hospitals. It requires well-trained technicians to locate electrodes, since all the electrodes have to be placed in the proper location. And it is time-consuming. All these reasons are making the system difficult to apply in real life. Therefore, a worthwhile EEG system with fewer channels or even one channel for estimating driver fatigue has to be a portable system that is cheaper, simpler, and easier to use.

Although many EEG-based methods have been proven to detect driver fatigue, the optimal method has not yet been determined. Furthermore, the EEG with more channels usually restricts its application in the detection of driver fatigue. Using the data from 12 subjects, the detection model for driver fatigue was developed with a single channel. Four types of entropies were deployed in this work: SE, FE, AE, and PE. The classification procedure was implemented by ten classifiers:* K*-Nearest Neighbors (KNN), SVM with linear kernel (LS), SVM with RBF kernel (RS), Gaussian Process (GP), Decision Tree (DT), RF, Multilayer Perceptron (MLP), AdaBoost (AB), Gaussian Naïve Bayes (GNB), and Quadratic Discriminant Analysis (QDA). The aims of the present study are to determine the optimal combination of feature, classifier, and channel that can be effective in portable application with a single channel.

The rest of the paper is organized as follows: Section 2 describes the proposed methodology. Results and discussion are reported in Section 3. Conclusion is reported in Section 4.

## 2. Materials and Methods

### 2.1. Subjects

Twelve university students (men, 19–24 years) participated in this experiment. All the subjects were asked to be out of any type of stimulus like alcohol, medicine, tea, or coffee before and during the experiment. Before the experiment, they practiced the driving task for several minutes to become acquainted with the experimental procedures and purposes. All experimental procedures were performed using a static driving simulator in a software-controlled environment. This work was approved by Academic Ethics Committee of Jiangxi University of Technology.

### 2.2. Experiment

**Figure 1:**
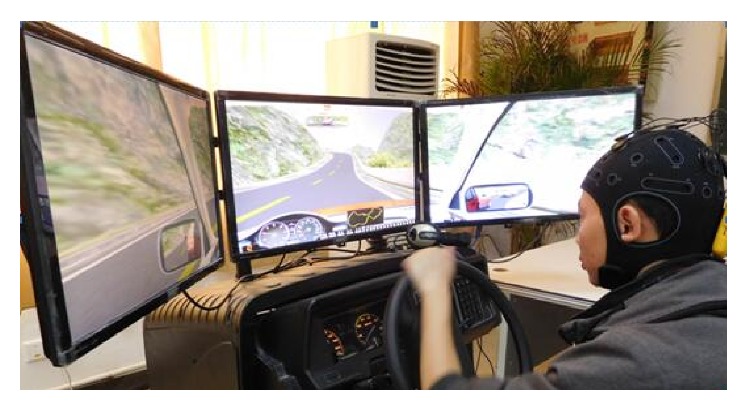
Snapshot of the experimental setup.

The experimental setup of the work is based on our previous work. A sustained-attention driving task was performed by each subject on a static driving simulator (The ZY-31D car driving simulator, produced by Peking ZhongYu Co., Ltd.) with a wide screen composed of three 24-inch monitors shown as in Figure 1. On the screen, a customized version of the Peking ZIGUANGJIYE software ZG-601 (Car Driving Simulation Teaching System V9.2) was shown. The driving environment selected for this study was a highway with low traffic density and the driving task was started at 9 a.m. After the 5-minute practice session, each subject was given a break of 10 min away from the simulator and was allowed to have unconstrained movement within the laboratory. Then they commenced their about 1-2 hours of driving after a quick check of all instrumentation.

### 2.3. Data Recording and Preprocessing

**Figure 2:**
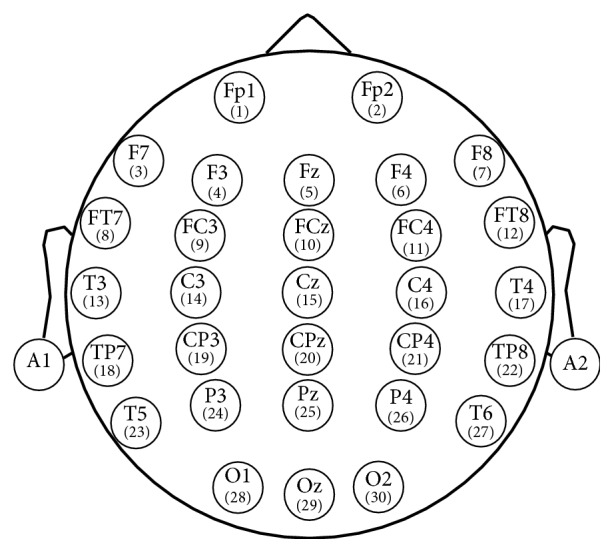
Electrodes position according to International 10-20 System standards.

First, when the subjects had been driving for 20 minutes, the last 5-minute recorded EEG signal was labeled as normal state; second, when the continuous driving procedure lasted 60–120 minutes until the questionnaire results show the subject was in driving fatigue, obeying Lee's subjective fatigue scale and Borg's CR-10 scale [29, 30], the last 5-minute recorded EEG signal was labeled as fatigue state. All channel data were referenced to two electrically linked mastoids at A1 and A2, digitized at 1000 Hz from a 32-channel electrode cap (including 30 effective channels and 2 reference channels) based on the International 10-20 System (Figure 2).

After the EEG signals acquisition, the main steps of data preprocessing were carried out by the Scan 4.3 software of Neuroscan. The raw signals were first filtered by a 50 Hz notch filter and a 0.15 Hz to 45 Hz band-pass filter was used. Then 5-minute EEG signals from 30 channels were sectioned into 1-s epochs, resulting in 300 epochs. With the 12 subjects, a total of 3600 epochs of dataset were formed for the normal state and another 3600 epochs for the fatigue state.

### 2.4. Feature Extraction

In recent years, various entropies have been expanded in several different fields [31]. As the nonlinear parameters can quantify the complexity of a time series, it can be used to evaluate the nonlinear, unstable EEG signals [32]. PE is calculated by applying the Shannon function to the normalized power spectrum, and the calculation algorithm is as described in literature [33]. AE, proposed by Pincus [34], is calculated in time domain without phase space reconstruction of the signal. Similar to AE, SE is proposed by Richman and Moorman [35]. The calculation algorithm of AE and SE is defined clearly as described in literature [36]. FE can get stable results for different parameters and offers better noise resistance, defined clearly as described in literature [37].

In the above four types of entropies, AE, SE, and FE have parameters, *m* and *r*, which are the dimensions of phase space and similarity tolerance, respectively. Generally, too larger of *r* will lead to a loss of useful information. However, if *r* is underestimated, the sensitivity to noise will be increased significantly. In the present study, *m* = 2 while *r* = 0.2*∗*SD, where SD denotes the standard deviation of the time series according to literature [38].

For optimizing the detection quality, the features were normalized for each subject by scaling between −1 and 1.

### 2.5. Classification

Since there is no uniform classification method suitable for all subjects and all applications, usually it may be useful to test multiple methods. In this work, I have used ten classifiers, namely, KNN, LS, RS, GP, DT, RF, MLP, AB, GNB, and QDA. They are briefly explained below.

#### 2.5.1. KNN

Neighbors-based classification does not construct a general model but simply compares instances of features of the training data. KNN is a supervised learning technique where a new instance is classified based on the closest training samples present in the feature space [39]. KNN implements learning based on the *k*-Nearest Neighbors of each query point, where *k* is 5 in this study.

#### 2.5.2. SVM

In the case of nonlinear classification, kernels, such as radial basis functions (RBF), are used to map the data into a higher dimensional feature space in which a linear separating hyperplane could be found [40]. When the number of samples is less than the number of features, nonlinear learning methods do not significantly affect the results and it may be better to simply use linear learning method. So SVM with linear kernel (LS) and SVM with RBF kernel (RS) were both chosen as the classifier in this work.

When training an SVM classifier with the RBF kernel, two parameters must be considered: *c* and *γ*. A lower *c* makes the decision surface smooth, while a higher *c* aims at classifying all training examples correctly. *γ* defines how much influence a single training example has. In this study, *γ* = 2 and *c* = 1.

#### 2.5.3. GP

The GP Classifier implements Gaussian Processes for classification purposes, more specifically for probabilistic classification [41].

#### 2.5.4. DT

DT is a nonparametric supervised learning method used for classification [42]. DT creates a series of binary decisions on the features which best distinguishes classes. The maximum depth of the tree is 10 in this work.

#### 2.5.5. RF

RF fits a number of Decision Tree classifiers on various subdatasets and averages predicted accuracy [43]. In this work, the maximum depth of the tree is 10 and the number of trees in the forest is 10.

#### 2.5.6. MLP

MLP trains using gradient descent and the gradients are calculated using Backpropagation (BP) [44].

#### 2.5.7. AB

AB classifier begins by fitting a classifier on the raw dataset and then fits additional copies of the classifier on the same dataset where the weights of incorrectly classified instances are adjusted [45].

#### 2.5.8. GNB

Naive Bayes method is based on applying Bayes' theorem with the “naive” assumption [46]. The likelihood in GNB of the features is assumed to be Gaussian.

#### 2.5.9. QDA

QDA searches for a linear combination of features which statistically best distinguishes objects in different classes from each other [47]. QDA classifier has a quadratic decision boundary.

### 2.6. Performance Metrics

For developing a new detector and estimating its potential application performance, it is very important to examine properly the detection quality [48]. The leave-one-out (LOO) cross-validation approach is used to assess the performance of the system for driver fatigue detection. The total average accuracy based on some feature and the classifier is the average of the accuracy of all single channels based on the same feature and same classifier.

To provide an easier-to-understand method to measure the detection quality, the well-known performance indicators [43], including accuracy (Acc), sensitivity (Sn), and specificity (Sp), were described as follows:

(1) Sn=TPTP+FN,Sp=TNTN+FP,Acc=TP+TNTP+TN+FP+FN,

where TP (true positive) denotes the number of the data inputs that refer to fatigue state correctly classified as fatigue. FP (false positive) is the number of data inputs that refer to normal state classified as fatigue state. TN (true negative) is number of the data inputs that refer to normal state correctly classified as normal state. FN (false negative) is the data inputs that refer to fatigue state classified as normal state.

AUC illustrates the performance of a binary classifier system as its discrimination threshold is varied. It is created by plotting the fraction of true positives out of the positives (TPR = true positive rate) versus the fraction of false positives out of the negatives (FPR = false positive rate), at various threshold settings. TPR is also known as Sn, and FPR is one minus the Sp.

## 3. Results and Discussion

### 3.1. Comparison of Performances of Different Subjects

**Figure 3:**
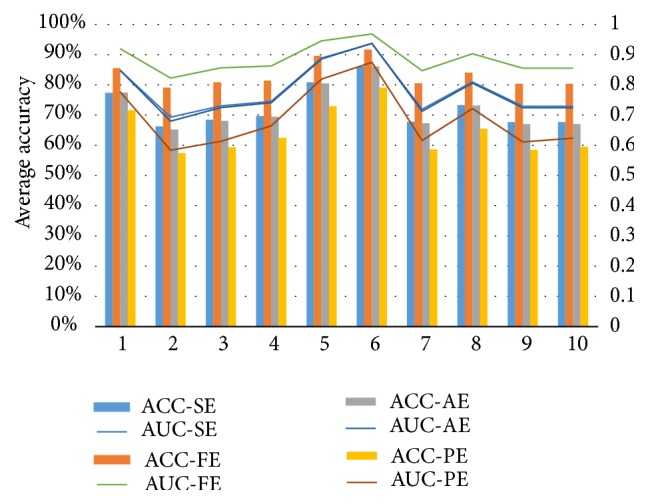
Comparison of performances of four features and ten classifiers. The left s vertical coordinate is for average accuracy (%) for 12 subjects, while the right vertical coordinate is for average AUC for 12 subjects. The horizontal coordinate is for classifier. 1–10 represent KNN, LS, RS, GP, DT, RF, MLP, AB, GNB, and QDA, respectively. ACC-SE, ACC-FE, ACC-AE, and ACC-PE represent accuracy with features SE, FE, AE, and PE, respectively. AUC-SE, AUC-FE, AUC-AE, and AUC-PE represent AUC with features SE, FE, AE, and PE, respectively.

As shown in Figure 3 and Table 1, the best average accuracy is produced in combination of FE + RF (where average accuracy is 91.7%) and the worst average accuracy is produced in combination of SE + LS (where average accuracy is 57.4%). It can be found that the best accuracies of Subject 1 and Subject 2 all occurred in the combination of FE + KNN while, for Subjects 3–12, best recognition rates all appear in the combination of FE + RF. The worst recognition rate of Subject 1 appears in combination of SE + LS, while, for Subjects 2, 5, 6, 7, 9, 11, and 12, it appears in the combination of PE + LS, and, for Subjects 3, 4, 8, and 10, it appears in the combination of PE + MLP. For all 12 subjects, the highest accuracy is 94.4% for Subject 9 with the combination of FE + RF, and the worst recognition rate (51.7%) also appeared in Subject 9 with the combination of SE + LS. This is an interesting phenomenon. For the same subject, using different methods, some subjects will have a particularly larger difference, and some may be less.

As for the AUC, there are similar results. The best AUC is produced in combination of FE + RF (where average AUC is 0.969) and the worst average accuracy is produced in combination of PE + LS (where average AUC is 0.584). For all 12 subjects, the highest AUC (0.983) appears in Subject 9, and the worst AUC (0.517) also appears in Subject 9. This is very similar to ACC.

Different subjects have different brain characteristics, so the EEG features are different. Different subjects using the same feature extraction method or the same classifier may have different performances. The result has two meanings, one is that it is possible to choose a combination that is subject-specific, which is different from the subjects using different combination, thus improving the recognition rate of each subject. Two is that subject-specific EEG feature can be distinguished from different subjects for identification or authentication of individual, that is, the EEG password or biometrics [49, 50].

### 3.2. Comparison of Four Feature Methods

From the above results, the combination of entropy and classifier improved the classification performance. Because the main purpose of my study is to find the optimal combination of feature and classifier based on a single EEG channel, in order to evaluate the performance influence on different entropy features, four types of entropy feature methods and ten classifiers were compared. Figure 3 shows the mean accuracy of generated features obtained from the four entropy methods based on EEG signals from all single channels of 12 subjects, using ten classifiers. From Figure 3, I can conclude that the classification accuracy of the combination of FE with any one of the classifiers is better than combination of the other feature methods with any one of the classifiers. Hence FE was selected as best feature in this work as it is robust and efficient. The detector of using FE + RF fusion method could present a better performance and robustness.

As shown in Table 2, the average accuracy was compared with 12 subjects based on different feature and classifier. The average accuracy based on FE feature was 83.5%, while the average accuracy based on PE feature was 64.4%. The highest mean Acc appeared at the combination of FE + RF, reaching 91.8%, while the worst mean Acc appeared at the combination of PE + LS, achieving 57.3%. These results are in agreement with the results of Section 3.1.

As shown in Table 3, the average AUC was compared with 12 subjects based on different feature and classifier. The average AUC based on FE feature was 0.885, while the average AUC based on PE feature was 0.689. The highest mean AUC occurred at the combination of FE + RF, reaching 0.969, while the worst mean AUC occurred at the combination of PE + LS, achieving 0.584. These results are also in agreement with the results of Section 3.1.

### 3.3. Comparison of Ten Classifiers

Overall, sorting from large to small of the average accuracy of ten classifiers based on four features and 12 subjects is RF∖DT∖KNN∖AB∖GP∖RS∖QDA∖MLP∖GNB∖LS. The sort of mean AUC is the same.

For 12 subjects, I used *k* = 1, 3, and 5 for KNN and found that *k* = 5 gave the best performance. It can be seen that KNN achieves the highest accuracy with 94.3% and AUC of 0.976 with FE feature. These accuracies are better than previous studies.

### 3.4. Comparison of Channels

For channel comparison, the performance of each channel is determined. In order to compare the performance of each channel, with average of 12 subjects, the four types of combinations were compared, including combination of the best features and the best classifier (FE + RF), combination of the best feature + the worst classifier (FE + LS), combination of the worst features and the best classifier (PE + RF), combination of the worst feature + classifier (PE + LS). It can be seen that the highest Acc of single channel is 96.6% at the combination of CP4 + FE + RF, which can fully meet the requirements of mobile computing. The worst Acc is only 55.2% at the combination of Cz + PE + LS.

**Figure 4:**
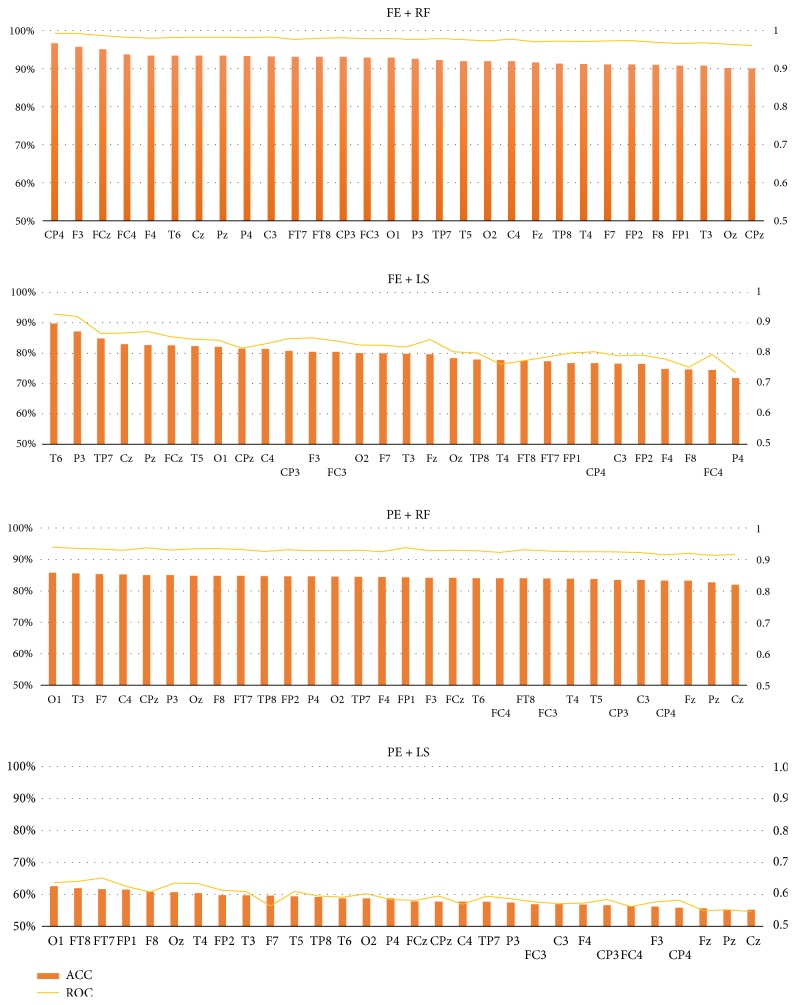
Comparison of all channels based on feature FE and classifier RF of 12 subjects. The left s vertical coordinate is for accuracy (%), while the right vertical coordinate is for AUC. The horizontal coordinate is for channel.

It can be seen from Figure 4 that all channels of the four combinations are sorted according to the Acc. The index is not the same order in the four combinations. For example, the best channel is CP4 at the combination of FE + RF, while the best channel is T6 at the combination of FE + LS, and the best channel is O1 at the combination of both PE + RF and PE + LS, indicating channel selection is related to feature extracted methods and classifier methods largely.

The result of AUC is very similar. The highest AUC of single channel is 0.993 at the combination of CP4 + FE + RF, while the worst AUC is only 0.545 at the combination of Cz + PE + LS.

In addition to the variation of different channels shown as in Figure 4, we are concerned about which part of brain regions these select channels locate over. So the selected electrodes in each subject were mapped onto their corresponding locations in the electrode cap. It can be seen that the distribution of top channels is much more scattered.

The above results demonstrated the system using a single channel could achieve very high accuracy in detecting driver fatigue, while reducing the decisive number of electrodes from 30 to 1. It is possible to use single channel for driver fatigue detection. The highest recognition rate in this work can be up to 96.6%, which is not the worst comparing with other research results.

Sort of channel is not related to hemisphere, and there is no significant correlation between brain areas. For each subject, the best channel is not the same.

For different analysis targets, using different features may have different impacts on the classification accuracy. In this paper I selected four entropies for comparison purpose. Figure 1 indicates that, for the same data source, the classification performances of the four entropies and ten classifiers are notably different. In my experiment paradigm, the combination of feature FE and classifier RF has the highest accuracy if single entropy is used as input. As see in Table 4, it is found that the classification performance of the proposed method was better than the other research using fewer channels of EEG signals; it is expected that the combination of feature FE, classifier RF, and channel CP4 can show better performance for fatigue forecast. Although the present study is based on the existing EEG data, the high performance of detection of driving fatigue by using of FE-based classification indicated well application on the real-time detection of driving fatigue. To realize real-time detection of driving fatigue, I only needed to record a single channel EEG signals when in fatigue state and normal state and then trained FE-based classification. Once the trained classification model is being saved, I could achieve real-time detection of driving fatigue and try to avoid traffic accidents through the alarm.

## 4. Conclusions

In this paper, an approach based on combination of four entropy features and ten classifiers is proposed to detect driver fatigue in an EEG-based system. Results also showed that it is a promising system to detect driver fatigue, achieving high success rates with only one electrode. The following was found: (1) It is possible to use a single channel for driver fatigue detection. The highest recognition rate in this work can be up to 96.6%, which has been able to meet the needs of real applications. (2) The best combination of channel + features + classifier of different subjects is not the same; that is to say, the best combination is subject-specific. (3) The impact of feature on the accuracy and AUC is larger. In this work, the Acc of FE as the feature is far greater than the Acc of PE as the feature. (4) The impact of the classifier on the Acc and AUC is larger. In this work, the Acc of classifier RF is the best, while classifier LS is the worst. (5) The impact of channel selection on the Acc and AUC is significant.

However, some limitations of this study are as follows: (1) the sample size was relatively small. To extend my research, in the future, I will increase the number of subjects to improve the validation of results and to classify more fatigue states such as severe fatigue. (2) The parameters of classifier did not carry out optimization, such as MLP and SVM which are very sensitive to parameters. It is also possible that there are no optimization parameters, so the performance for classifier MLP and SVM is not good. (3) In this work, only four kinds of entropy feature were compared, no more feature extraction methods, such as AR, wavelet, and spectrum.

It is hoped that these findings may have the generalizability to provide an effective approach for auxiliary diagnosis of driver fatigue, in order to maintain public health and avoid life threatening.

## Figures and Tables

**Table 1:** Optimal combination for different subjects.

Subject	Optimal combination	Highest Acc	AUC
1	FE + KNN	94.3%	0.976
2	FE + KNN	86.4%	0.929
3	FE + RF	93.4%	0.981
4	FE + RF	91.0%	0.969
5	FE + RF	92.6%	0.976
6	FE + RF	91.3%	0.974
7	FE + RF	91.4%	0.968
8	FE + RF	92.7%	0.981
9	FE + RF	94.4%	0.983
10	FE + RF	91.9%	0.975
11	FE + RF	90.5%	0.967
12	FE + RF	93.2%	0.979

**Table 2:** Comparison of mean accuracy (%) of combination of four features and ten classifiers.

Classifier	Feature				
	SE	FE	AE	PE	Mean ± SD
AB	73.2 ± 4.4	84.2 ± 3.6	72.9 ± 5.5	65.3 ± 6.1	73.9 ± 8.4
DT	80.6 ± 3.3	89.7 ± 2.9	80.2 ± 4.2	72.7 ± 5.7	80.8 ± 7.3
GP	69.5 ± 5.4	81.7 ± 4.1	69.0 ± 6.4	62.8 ± 6.0	70.8 ± 8.8
LS	66.0 ± 5.0	79.3 ± 4.4	64.8 ± 6.4	57.3 ± 7.2	66.9 ± 9.9
GNB	67.5 ± 6.0	80.5 ± 4.3	66.8 ± 7.1	58.2 ± 7.1	68.3 ± 10.1
KNN	77.3 ± 4.4	85.8 ± 3.4	77.4 ± 5.0	71.5 ± 7.6	78.0 ± 7.4
MLP	67.7 ± 5.5	80.7 ± 4.3	67.0 ± 6.8	58.5 ± 7.2	68.4 ± 10.0
QDA	67.5 ± 6.0	80.5 ± 4.3	66.8 ± 7.1	59.3 ± 7.1	68.5 ± 9.8
RF	85.9 ± 3.1	**91.8** ± **2.7**	85.9 ± 3.3	79.1 ± 9.3	85.7 ± 7.0
RS	68.3 ± 5.7	81.2 ± 4.1	67.9 ± 6.8	59.2 ± 6.8	69.1 ± 9.8
Mean ± SD	72.3 ± 8.1	83.5 ± 5.6	71.9 ± 9.0	64.4 ± 10.1	

Boldface indicates FE + RF is the optimal method.

**Table 3:** Comparison of mean AUC of combination of four features and ten classifiers.

Classifier	Feature				
	SE	FE	AE	PE	Mean ± SD
AB	0.808 ± 0.044	0.904 ± 0.027	0.804 ± 0.053	0.720 ± 0.080	0.809 ± 0.085
DT	0.886 ± 0.033	0.946 ± 0.025	0.883 ± 0.038	0.817 ± 0.060	0.883 ± 0.061
GP	0.743 ± 0.059	0.865 ± 0.037	0.736 ± 0.069	0.667 ± 0.077	0.753 ± 0.095
LS	0.690 ± 0.053	0.825 ± 0.055	0.674 ± 0.068	0.584 ± 0.098	0.693 ± 0.111
GNB	0.726 ± 0.063	0.857 ± 0.036	0.720 ± 0.073	0.609 ± 0.090	0.728 ± 0.111
KNN	0.847 ± 0.044	0.921 ± 0.025	0.847 ± 0.050	0.775 ± 0.099	0.848 ± 0.080
MLP	0.716 ± 0.063	0.850 ± 0.040	0.709 ± 0.075	0.615 ± 0.092	0.722 ± 0.109
QDA	0.726 ± 0.063	0.857 ± 0.036	0.720 ± 0.073	0.622 ± 0.090	0.731 ± 0.108
RF	0.936 ± 0.031	**0.969** ± **0.021**	0.937 ± 0.031	0.874 ± 0.111	0.929 ± 0.070
RS	0.0728 ± 0.062	0.859 ± 0.036	0.721 ± 0.074	0.610 ± 0.087	0.729 ± 0.111
Mean ± SD	0.780 ± 0.095	0.885 ± 0.057	0.775 ± 0.104	0.689 ± 0.132	

Boldface indicates FE + RF is the optimal method.

**Table 4:** Studies regarding driver fatigue detection using different types of entropy.

Research group	Feature method	EEG channels	Highest accuracy
Li et al. [14]	12 types of energy parameters	FP1 and O1	91.5%
Zhang et al. [27]	Approximate entropy	O1 and O2	96.5%
Khushaba et al. [51]	Fuzzy entropy	Fz, T8, and Oz	92.8%
Zhao et al. [52]	Sample entropy	F3	95.0%
This paper	Fuzzy entropy	CP4	96.6%

## References

[1] Lal S. K. L., Craig A. (2001). A critical review of the psychophysiology of driver fatigue. *Biological Psychology*.

[2] Saini V., Saini R. (2014). Driver drowsiness detection system and techniques: a review. *Computer Science and Information Technologies*.

[3] Dong Y., Hu Z., Uchimura K., Murayama N. (2011). Driver inattention monitoring system for intelligent vehicles: a review. *IEEE Transactions on Intelligent Transportation Systems*.

[4] Sahayadhas A., Sundaraj K., Murugappan M. (2012). Detecting driver drowsiness based on sensors: a review. *Sensors*.

[5] Jo J., Lee S. J., Park K. R., Kim I.-J., Kim J. (2014). Detecting driver drowsiness using feature-level fusion and user-specific classification. *Expert Systems with Applications*.

[6] Niu G., Wang C. (2014). Driver fatigue features extraction. *Mathematical Problems in Engineering*.

[7] Fu R., Wang H. (2014). Detection of driving fatigue by using noncontact emg and ecg signals measurement system. *International Journal of Neural Systems*.

[8] Ma J., Shi L., Lu B. (2014). An EOG-based vigilance estimation method applied for driver fatigue detection. *Neuroscience & Biomedical Engineering*.

[9] Wang H. Detection and alleviation of driving fatigue based on EMG and EMS/EEG using wearable sensor.

[10] Correa A. G., Orosco L., Laciar E. (2014). Automatic detection of drowsiness in EEG records based on multimodal analysis. *Medical Engineering & Physics*.

[11] Mu Z., Hu J., Yin J. (2016). Driving fatigue detecting based on EEG signals of forehead area. *International Journal of Pattern Recognition and Artificial Intelligence*.

[12] Yin J., Hu J., Mu Z. (2016). Developing and evaluating a mobile driver fatigue detection network based on electroencephalograph signals. *Healthcare Technology Letters*.

[13] Fu R., Wang H., Zhao W. (2016). Dynamic driver fatigue detection using hidden Markov model in real driving condition. *Expert Systems with Applications*.

[14] Li W., He Q.-C., Fan X.-M., Fei Z.-M. (2012). Evaluation of driver fatigue on two channels of EEG data. *Neuroscience Letters*.

[15] Wali M. K., Murugappan M., Ahmmad B. (2013). Wavelet packet transform based driver distraction level classification using EEG. *Mathematical Problems in Engineering*.

[16] Simon M., Schmidt E. A., Kincses W. E. (2011). Eeg alpha spindle measures as indicators of driver fatigue under real traffic conditions. *Clinical Neurophysiology*.

[17] Charbonnier S., Roy R. N., Bonnet S., Campagne A. (2016). EEG index for control operators' mental fatigue monitoring using interactions between brain regions. *Expert Systems with Applications*.

[18] Apker G., Lance B., Kerick S., McDowell K. Combined linear regression and quadratic classification approach for an EEG-based prediction of driver performance.

[19] Hajinoroozi M., Mao Z., Jung T., Lin C., Huang Y. (2016). EEG-based prediction of driver's cognitive performance by deep convolutional neural network. *Signal Processing: Image Communication*.

[20] Zhao C., Zheng C., Zhao M., Liu J., Tu Y. (2011). Automatic classification of driving mental fatigue with EEG by wavelet packet energy and KPCA-SVM. *International Journal of Innovative Computing, Information and Control*.

[21] Kong W., Lin W., Babiloni F., Hu S., Borghini G. (2015). Investigating driver fatigue versus alertness using the granger causality network. *Sensors*.

[22] Zhao C., Zhao M., Yang Y., Gao j., Rao N., Lin P. (2016). The Reorganization of Human Brain Networks Modulated by Driving Mental Fatigue. *IEEE Journal of Biomedical and Health Informatics*.

[23] Liang S.-F., Kuo C.-E., Hu Y.-H., Pan Y.-H., Wang Y.-H. (2012). Automatic stage scoring of single-channel sleep EEG by using multiscale entropy and autoregressive models. *IEEE Transactions on Instrumentation and Measurement*.

[24] Acharya U. R., Molinari F., Sree S. V., Chattopadhyay S., Ng K.-H., Suri J. S. (2012). Automated diagnosis of epileptic EEG using entropies. *Biomedical Signal Processing and Control*.

[25] Xiong Y., Gao J., Yang Y., Yu X., Huang W. (2016). Classifying driving fatigue based on combined entropy measure using EEG signals. *International Journal of Control and Automation*.

[26] Chai R., Naik G., Nguyen T. N. (2016). Driver fatigue classification with independent component by entropy rate bound minimization analysis in an EEG-based system. *IEEE Journal of Biomedical and Health Informatics*.

[27] Zhang C., Wang H., Fu R. (2014). Automated detection of driver fatigue based on entropy and complexity measures. *IEEE Transactions on Intelligent Transportation Systems*.

[28] Kar S., Bhagat M., Routray A. (2010). EEG signal analysis for the assessment and quantification of driver's fatigue. *Transportation Research Part F: Traffic Psychology and Behaviour*.

[29] Lee K. A., Hicks G., Nino-Murcia G. (1991). Validity and reliability of a scale to assess fatigue. *Psychiatry Research*.

[30] Borg G. (1990). Psychophysical scaling with applications in physical work and the perception of exertion. *Scandinavian Journal of Work, Environment & Health*.

[31] Ellis R. S. (2012). *Entropy, Large Deviations, and Statistical Mechanics*.

[32] Azarnoosh M., Motie Nasrabadi A., Mohammadi M. R., Firoozabadi M. (2011). Investigation of mental fatigue through EEG signal processing based on nonlinear analysis: symbolic dynamics. *Chaos, Solitons & Fractals*.

[33] Kannathal N., Choo M. L., Acharya U. R., Sadasivan P. K. (2005). Entropies for detection of epilepsy in EEG. *Computer Methods and Programs in Biomedicine*.

[34] Pincus S. M. (1991). Approximate entropy as a measure of system complexity. *Proceedings of the National Academy of Sciences of the United States of America*.

[35] Richman J. S., Moorman J. R. (2000). Physiological time-series analysis using approximate and sample entropy. *American Journal of Physiology—Heart and Circulatory Physiology*.

[36] Song Y., Crowcroft J., Zhang J. (2012). Automatic epileptic seizure detection in EEGs based on optimized sample entropy and extreme learning machine. *Journal of Neuroscience Methods*.

[37] Xiang J., Li C., Li H. (2015). The detection of epileptic seizure signals based on fuzzy entropy. *Journal of Neuroscience Methods*.

[38] Yentes J. M., Hunt N., Schmid K. K., Kaipust J. P., McGrath D., Stergiou N. (2013). The appropriate use of approximate entropy and sample entropy with short data sets. *Annals of Biomedical Engineering*.

[39] Li X., Hu B., Sun S., Cai H. (2016). EEG-based mild depressive detection using feature selection methods and classifiers. *Computer Methods & Programs in Biomedicine*.

[40] Mu Z., Hu J., Min J. (2016). EEG-based person authentication using a fuzzy entropy-related approach with two electrodes. *Entropy*.

[41] Zhong M., Lotte F., Girolami M., Lécuyer A. (2008). Classifying EEG for brain computer interfaces using Gaussian processes. *Pattern Recognition Letters*.

[42] Polat K., Güneş S. (2007). Classification of epileptiform EEG using a hybrid system based on decision tree classifier and fast Fourier transform. *Applied Mathematics & Computation*.

[43] Fraiwan L., Lweesy K., Khasawneh N., Wenz H., Dickhaus H. (2012). Automated sleep stage identification system based on time-frequency analysis of a single EEG channel and random forest classifier. *Computer Methods & Programs in Biomedicine*.

[44] Orhan U., Hekim M., Ozer M. (2011). EEG signals classification using the K-means clustering and a multilayer perceptron neural network model. *Expert Systems with Applications*.

[45] Sabeti M., Katebi S., Boostani R. (2009). Entropy and complexity measures for EEG signal classification of schizophrenic and control participants. *Artificial Intelligence in Medicine*.

[46] Do L.-N., Yang H.-J., Kim S.-H., Lee G.-S., Kim S.-H. (2015). A multi-voxel-activity-based feature selection method for human cognitive states classification by functional magnetic resonance imaging data. *Cluster Computing*.

[47] Vidaurre C., Schlögl A., Cabeza R., Scherer R., Pfurtscheller G. (2007). Study of on-line adaptive discriminant analysis for EEG-based brain computer interfaces. *IEEE Transactions on Biomedical Engineering*.

[48] Azar A. T., El-Said S. A. (2014). Performance analysis of support vector machines classifiers in breast cancer mammography recognition. *Neural Computing and Applications*.

[49] Hu J., Mu Z., Wang P. (2015). Multi-feature authentication system based on event evoked electroencephalogram. *Journal of Medical Imaging and Health Informatics*.

[50] Bao X., Wang J., Hu J. Method of individual identification based on electroencephalogram analysis.

[51] Khushaba R. N., Kodagoda S., Lal S., Dissanayake G. (2011). Driver drowsiness classification using fuzzy wavelet-packet-based feature-extraction algorithm. *IEEE Transactions on Biomedical Engineering*.

[52] Zhao X., Xu S., Rong J., Zhang X. (2013). Discriminating threshold of driving fatigue based on the electroencephalography sample entropy by receiver operating characteristic curve analysis. *Xinan Jiaotong Daxue Xuebao/Journal of Southwest Jiaotong University*.

